# High-throughput detection of eukaryotic parasites and arboviruses in mosquitoes

**DOI:** 10.1242/bio.058855

**Published:** 2021-07-22

**Authors:** Matthew V. Cannon, Haikel N. Bogale, Devika Bhalerao, Kalil Keita, Denka Camara, Yaya Barry, Moussa Keita, Drissa Coulibaly, Abdoulaye K. Kone, Ogobara K. Doumbo, Mahamadou A. Thera, Christopher V. Plowe, Mark A. Travassos, Seth R. Irish, Joshua Yeroshefsky, Jeannine Dorothy, Brian Prendergast, Brandyce St. Laurent, Megan L. Fritz, David Serre

**Affiliations:** 1 Institute for Genome Sciences, Department of Microbiology and Immunology, University of Maryland School of Medicine, Baltimore, MD 21201, USA; 2 Programme National de Lutte contre le Paludisme, Guinea; 3 Malaria Research and Training Center, University Science, Techniques and Technologies of Bamako, Mali; 4 Malaria Research Program, Department of Medicine, Center for Vaccine Development and Global Health, University of Maryland School of Medicine, Baltimore, MD 21201, USA; 5 U.S. President's Malaria Initiative and Entomology Branch, Division of Parasitic Diseases and Malaria, Center for Global Health, Centers for Disease Control and Prevention, Atlanta, GA 30333, USA; 6 Department of Entomology, University of Maryland College Park, College Park, MD 20742, USA; 7 Mosquito Control Program, Maryland Department of Agriculture, Annapolis, MD 21401, USA; 8 Laboratory of Malaria and Vector Research, National Institute of Allergy and Infectious Diseases, Rockville, MD 20852, USA

**Keywords:** Vector-borne pathogens, Eukaryotic parasites, Arbovirus, Surveillance, Zoonosis, Emerging pathogens

## Abstract

Vector-borne pathogens cause many human infectious diseases and are responsible for high mortality and morbidity throughout the world. They can also cause livestock epidemics with dramatic social and economic consequences. Due to its high costs, vector-borne disease surveillance is often limited to current threats, and the investigation of emerging pathogens typically occurs after the reports of clinical cases. Here, we use high-throughput sequencing to detect and identify a wide range of parasites and viruses carried by mosquitoes from Cambodia, Guinea, Mali and the USA. We apply this approach to individual *Anopheles* mosquitoes as well as pools of mosquitoes captured in traps; and compare the outcomes of this assay when applied to DNA or RNA. We identified known human and animal pathogens and mosquito parasites belonging to a wide range of taxa, as well as DNA sequences from previously uncharacterized organisms. Our results also revealed that analysis of the content of an entire trap could be an efficient approach to monitor and identify rare vector-borne pathogens in large surveillance studies. Overall, we describe a high-throughput and easy-to-customize assay to screen for a wide range of pathogens and efficiently complement current vector-borne disease surveillance approaches.

## Abstract (precis)

**Summary:** We describe a surveillance technique enabling the detection of known and previously uncharacterized pathogens present in mosquitoes. This approach could reduce the costs of vector-borne disease monitoring and provide early warning of emerging diseases.

## INTRODUCTION

Different arthropods can, during a blood feeding, transmit viruses, protists and helminths to humans (Ecker et al., 2005). These organisms cause some of the most prevalent human infectious diseases, including malaria, dengue, schistosomiasis or Chagas disease, and are responsible for more than 700,000 human deaths worldwide every year [WHO, 2017; Institute of Medicine (US) Forum on Microbial Threats, 2008; Collaborators, 2018]. Vector-borne diseases are also responsible for some of the most alarming recent epidemics in the western hemisphere, either due to the emergence of new pathogens (e.g. Zika; Gorshkov et al., 2018; Liu et al., 2019), the reemergence of historically important pathogens (e.g. Yellow Fever; Higuera and Ramirez, 2019) or the expansion of diseases beyond their historical ranges (e.g. West Nile; Sejvar, 2016; and Chikungunya; Higuera and Ramirez, 2019). In addition to this burden on human health, many vector-borne diseases affect domesticated animals (e.g. heartworms; McCall et al., 2008; Otranto et al., 2013), livestock (e.g. Theileriosis; Nene et al., 2016; Gachohi et al., 2012) and wild animals (e.g. avian malaria; Clark et al., 2014; Lapointe et al., 2012). Some of these animal diseases have dramatic economic consequences in endemic areas (Nene et al., 2016; Gachohi et al., 2012), while others are zoonotic diseases, further affecting human populations (Cox-Singh et al., 2008; Daneshvar et al., 2018; White, 2008; Brasil et al., 2017; McCarthy and Moore, 2000; Cutler et al., 2010).

Efficient vector-borne disease surveillance is critical for reducing disease transmission and preventing outbreaks. Past elimination campaigns for vector-borne diseases, usually targeting a specific human pathogen, have often relied on entomological approaches such as widespread insecticide spraying and disruption of larval habitats (Benelli and Beier, 2017; Walker and Lynch, 2007). To be successful, such efforts need to be guided by detailed knowledge of the parasites' and vectors' distributions. Unfortunately, current entomological surveillance approaches are extremely resource-intensive: the collection of samples is time consuming and requires trained personnel, vector species identification is laborious, and the detection of pathogens is expensive since hundreds of mosquitoes typically need to be screened to identify a few infected ones. Consequently, public health officials and vector biologists typically focus on monitoring only a few specific pathogens associated with the most current threats. These constraints are particularly problematic as they hamper the early detection of emerging pathogens and vector surveillance is often implemented in response to reports of clinical cases rather than preventively.

We have recently described a sequencing-based method using amplicon sequencing to detect known and previously uncharacterized eukaryotic parasites from biological samples in a high-throughput and cost-efficient manner (Cannon et al., 2018). Here, we present the application of this approach to characterize a wide-range of eukaryotic parasites and arboviruses from more than 900 individual *Anopheles* mosquitoes collected in Cambodia, Guinea and Mali, as well as from 25 pools of mosquitoes captured in CDC CO_2_-baited light traps in Maryland, USA. We also compare the performance of the assay when screening DNA and RNA from the same samples. Overall, our study demonstrates how this sequencing-based assay could significantly improve monitoring of human and animal vector-borne pathogens.

## RESULTS

### Amplicon sequencing for high-throughput characterization of microorganisms in mosquitoes

**Fig. 1.**
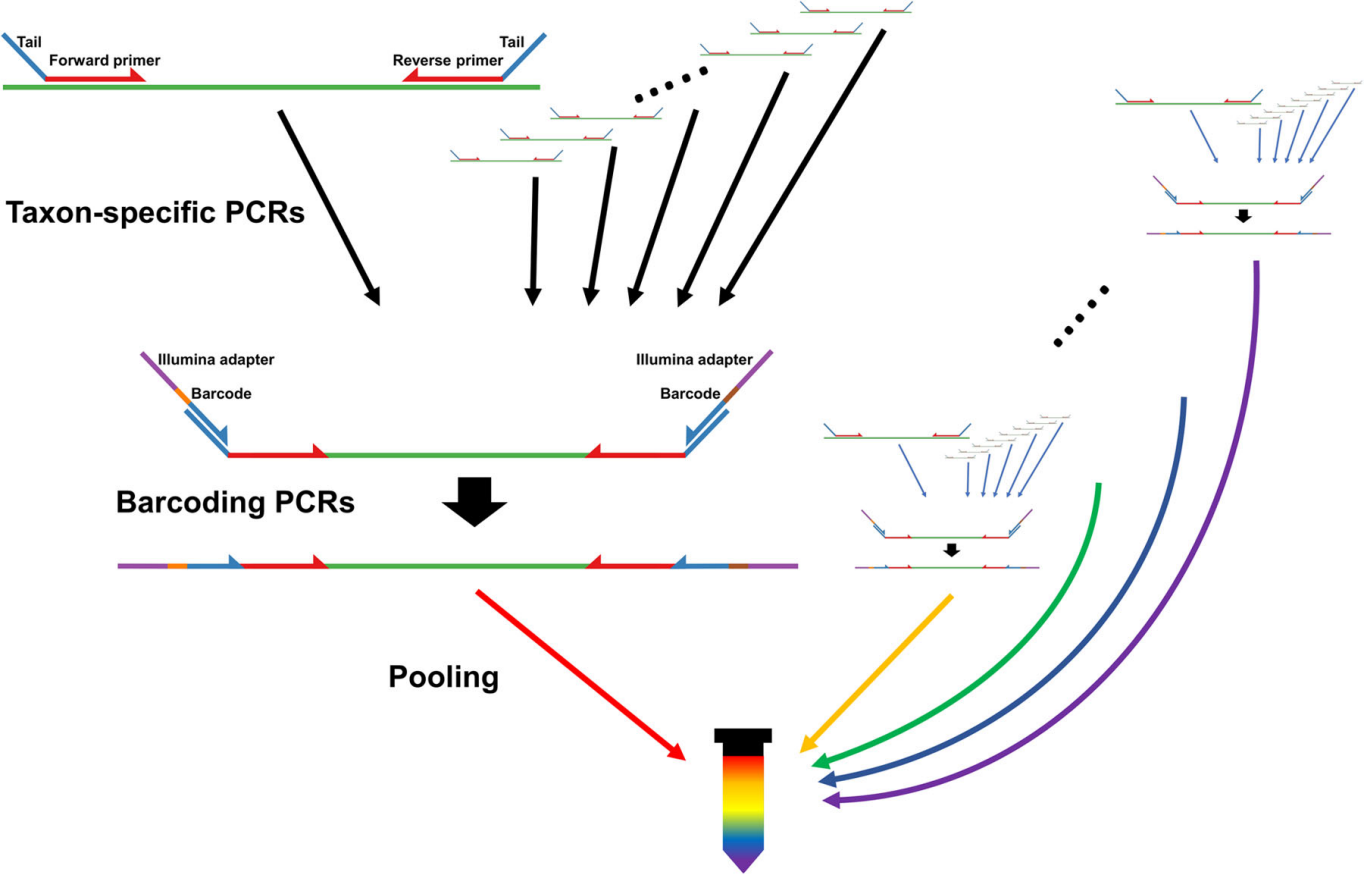
**Overview of the sequencing-based assay.** To create libraries for amplicon sequencing, we amplify each sample separately with tailed primers targeting each group of interest (Table 1). We then pool amplicons from each PCR by sample and perform a second amplification to incorporate a sample barcode and the Illumina adapter sequences. After the barcoding PCR, we pool all samples together before sequencing.

We analyzed 265 *Anopheles* mosquitoes collected in Cambodia, 665 *Anopheles* mosquitoes collected in Guinea and Mali as well as the content of 25 light traps, each containing 50–291 mosquitoes, collected in Maryland, USA. We screened each sample for a wide range of eukaryotic parasites using ten primer sets designed to amplify DNA from all species of taxa known to include human parasites: Apicomplexans, Kinetoplastids, Parabasalids, nematodes, Platyhelminthes and Microsporidians (Table S1). We also screened RNA extracted from the individual African *Anopheles* and from the pools of mosquitoes from Maryland for flaviviruses (see Materials and Methods; Table S1). After taxon-specific amplification, we pooled all PCR products generated from the same mosquito together, barcoded them and sequenced all libraries to generate an average of 12,703 paired-end reads per sample (Fig. 1). After merging read pairs, stringent quality filters and removal of the products of off-target amplification (e.g. *Anopheles* and bacteria DNA sequences), we obtained 61,177 unique DNA sequences, each represented by ten reads or more, and accounting in total for 6,796,105 reads (Table S2). These sequences were amplified with all primers and from a total of 185 samples: 42 out of 265 Cambodian mosquitoes (16%), 120 out of 665 African mosquitoes (18%), and 23 out of the 25 pools (92%) of mosquitoes collected in Maryland were positive for at least one of the taxa tested. On average, each sequence was supported by 1306 reads per sample (range: 10–43,440). By contrast, out of 176 negative controls, only 12 (7%) yielded any sequence from the targeted taxa and those were represented by 213 reads on average (range: 10–3539).

**Table 1.**
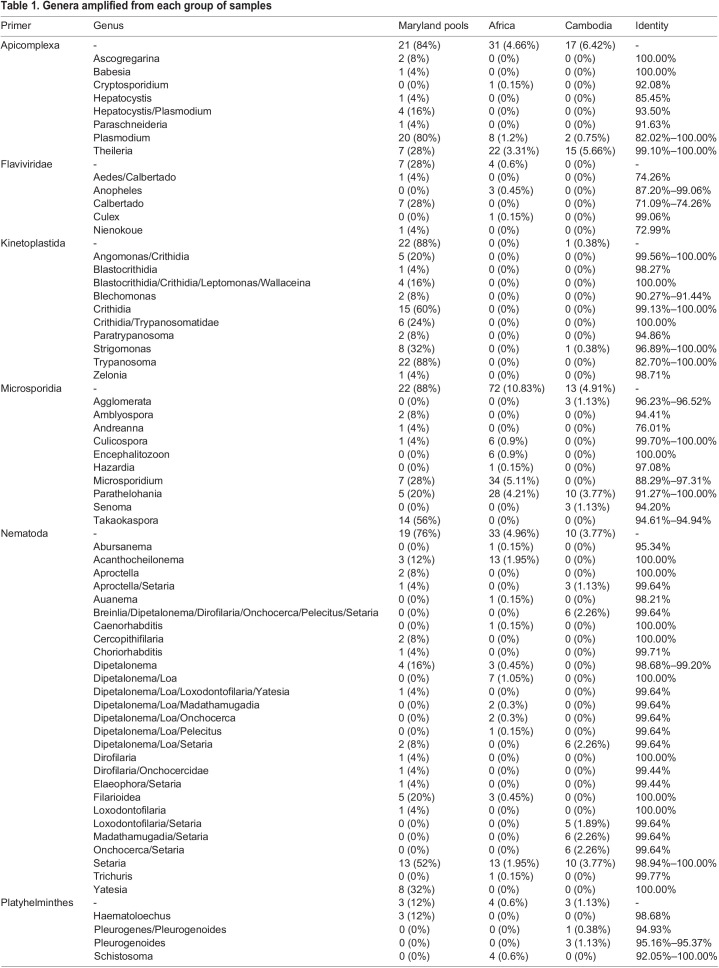
Genera amplified from each group of samples

### Identification of eukaryotic parasites

We retrieved DNA sequences identical to sequences previously amplified from *Theileria* parasites from 22 African and 15 Cambodian mosquitoes, as well as from seven of the Maryland traps. *Theileria* sequences were successfully amplified with both the Apicomplexa and Eimeronia primer pairs. All samples positive for *Theileria* with the Eimeronia primers were also positive with the Apicomplexa primers. On the other hand, the Eimeronia primers provided sufficient information to assign each sequence to a single species, while the sequences amplified with Apicomplexa primers were unable to differentiate among the *Theileria* species (see also below). We detected sequences identical to *Plasmodium falciparum* in eight African samples and two Cambodian samples, while sequences most similar (82.0%–99.5% identity) to bird *Plasmodium* species were amplified from 20 of the 25 traps in Maryland (Table 1). We also amplified a sequence that was identical to several *Babesia* species (100% identity) in one trap by two different primer pairs. Finally, we detected DNA from a known apicomplexan parasite of mosquitoes, *Ascogregarina barretti* (Siegel et al., 1992), in two of the traps.

From all individual mosquitoes, only one Cambodian *Anopheles* yielded a Kinetoplast sequence that was most similar to *Strigomonas culicis* (96.9% identity). By contrast, 22 of the traps were positive for Kinetoplasts, yielding sequences similar to sequences from *Angomonas*, *Blastocrithidia*, *Blechomonas*, *Crithidia*, *Leptomonas*, *Paratrypanosoma*, *Strigomonas*, *Trypanosoma*, *Trypanosomatidae*, *Wallaceina* or *Zelonia* (with 90.3%–100% identity, except for one sequence that matched *Trypanosoma theileri* at 82.7% identity) (Table 1).

Many sequences were amplified using the Microsporidia primers: 72 African mosquitoes were positive with sequences similar or identical to *Culicospora*, *Encephalitozoon*, *Hazarida*, *Microsporidium* and *Parathelohania* (88.3%–100% identity), while 13 Cambodian samples yielded sequences similar or identical to *Agglomerata*, *Parathelohania* and *Senoma* (91.3%–100% identity) (Table 1). Twenty-two traps also yielded Microsporidia sequences closely matching those of *Amblyospora*, *Andreanna*, *Culicospora*, *Microsporidium*, *Parathelohania* and *Takaokaspora* (with 76.0%–100% identity).

Regarding parasites from the Parabasalia group, four African mosquitoes were positive for *Tetratrichomona*, *Trichomonas* or *Tritrichomonas* with high sequence similarity (94.8%–100%) while a single Cambodian mosquito was positive for *Trichomitus* (98.7% identity). No Parabasalia were detected in the Maryland traps.

We detected Platyhelminthes sequences in four African mosquitoes, all similar to *Schistosoma mansoni* (92.1%–100% identity). Three Cambodian mosquitoes yielded sequences most similar to those of either *Pleurogenoides or Pleurogenes* (94.9%–95.4% identity). Three traps in Maryland were positive for Platyhelminthes, with sequences most similar to *Haematoloechus* (98.7% identity).

The taxonomic resolution of the nematode primers was lower than that of the other taxon-specific primer pairs and the amplified sequences often matched multiple species (or even genera). We amplified nematode sequences from 33 African *Anopheles*, including sequences most similar to *Abursanema*, *Acanthocheilonema*, *Auanema*, *Caenorhabditis*, *Dipetalonema*, *Filarioidea*, *Loa*, *Loxodontofilaria*, *Madathamugadia*, *Onchocerca*, *Pelecitus*, *Setaria* or *Trichuris*, although the sequence similarity (95.3–100%) clearly indicated that, in some cases, the exact identity of the species was unknown (see also below). Ten Cambodian mosquitoes were positive for *Setaria digitata* (100% identity) while other mosquitoes yielded sequences that matched *Setaria* and one or more of the following genera: *Aproctella*, *Breinlia*, *Dipetalonema*, *Dirofilaria*, *Loa*, *Loxodontofilaria*, *Madathamugadia*, *Onchocerca*, *Pelecitus*. Nineteen different traps from Maryland produced nematode sequences with particularly high read counts of *Setaria*, *Yatesia* and *Dirofilaria* sequences (98.9%–100% identity). Other genera detected in the traps included *Acanthocheilonema*, *Aproctella*, *Cercopithifilaria*, *Choriorhabditis*, *Dipetalonema*, *Elaeophora*, *Filarioidea*, *Loa*, *Loxodontofilaria*, *Onchocercidae*.

Overall, using this single assay, we screened over 3500 mosquitoes from three geographic locations and identified DNA sequences from numerous microorganisms encompassing six classes, 12 orders and 23 families (Table 1).

### Identification of flaviviruses in mosquitoes

**Fig. 2.**
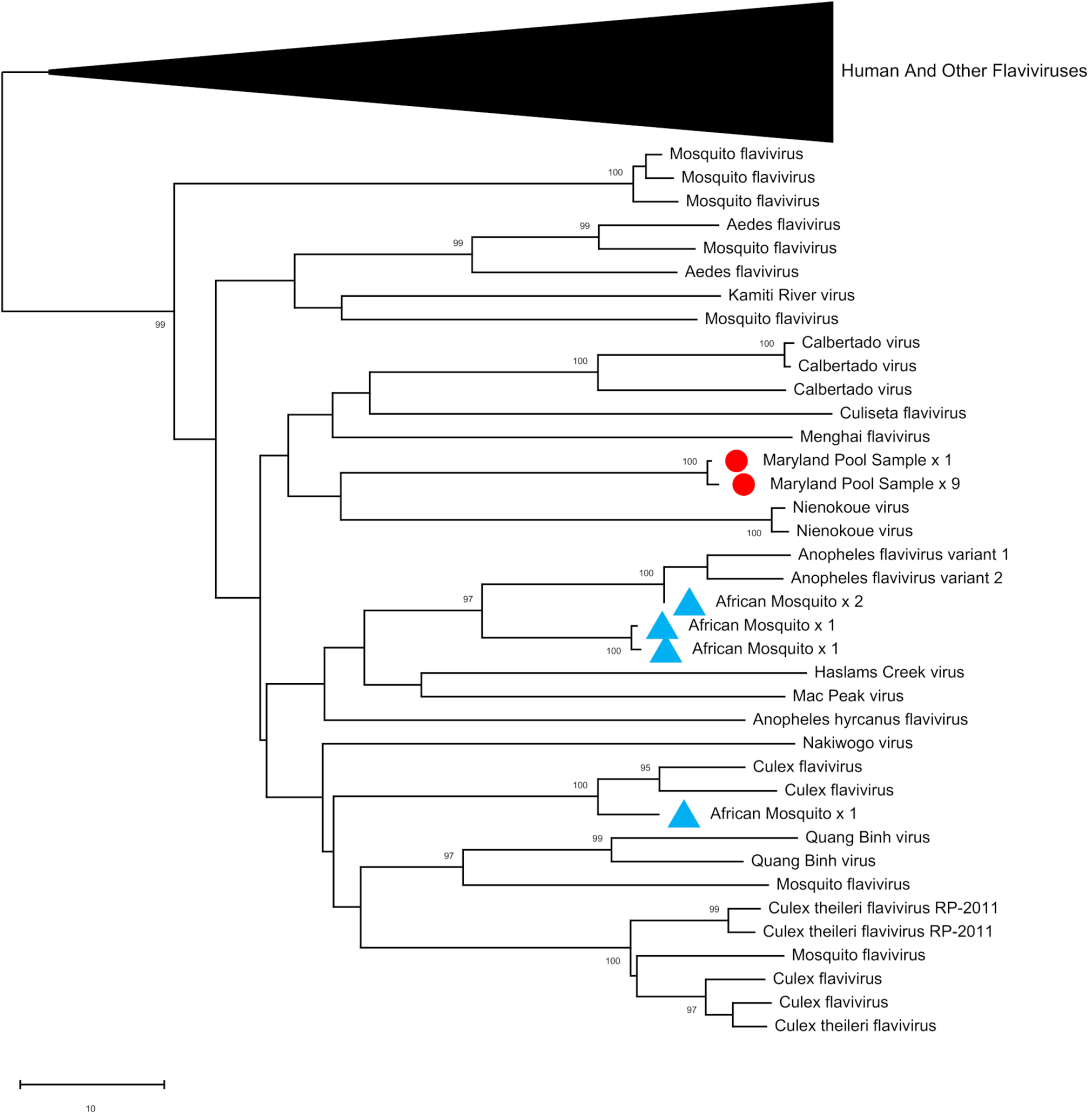
**Phylogenetic analysis of flavivirus sequences amplified from mosquitoes.** The neighbor-joining tree shows the relationships between the flavivirus sequences amplified from mosquito pools from Maryland (red circles) and from individual African mosquitoes (blue triangles). The number of positive samples is provided for each sequence. Phylogenetic tree without the compressed branch is available in Fig. S1 and alignment is available as Dataset 1.

To detect and identify flaviviruses, we used a primer pair predicted *in silico* to amplify a wide range of flaviviruses, including all known human pathogens (Patel et al., 2013), and we validated that these primers successfully amplified cDNA generated from West Nile, Zika and Dengue viruses. Out of 665 individual African mosquitoes, three were positive for viruses most similar to *Anopheles flavivirus variants 1* and *2* (87.2%–99.1% identity) (Fig. 2; Fig. S1) and one was positive for a virus similar to *Culex flavivirus* (99.1% identity). Seven Maryland traps (24%) were positive for flaviviruses. These viruses were most similar to the *Calbertado* and *Nienokoue* flaviviruses, although the percent identity was very low (71.1%–74.3%) and they clearly separated from those viruses in phylogenetic analysis (Fig. 2; Fig. S1). These sequences possibly derive from flaviviruses that have not yet been characterized but, since that they cluster with other mosquito flaviviruses (Fig. 2; Fig. S1), it is likely that they represent mosquito-infecting viruses rather than new human pathogens.

One limitation of our study of viral RNA is that the mosquitoes collected in Maryland, USA were, as typical in many entomological surveys, stored at room temperature upon collection which might have affected RNA preservation. To rigorously assess the stability of viral and mosquito RNA, we analyzed *Culex* mosquitoes from a laboratory colony known to be infected with *Culex flavivirus*. Pools of five mosquitoes were stored at room temperature for up to four weeks after collection, with and without preservative (see Materials and Methods for details). After RNA extraction and cDNA synthesis, we determined the amount of mosquito and virus RNA amplifiable using real-time PCR. Without preservative, the mosquito RNA was largely degraded after two weeks (detectable in only one of three replicates) and undetectable after 4 weeks (Fig. S2). By comparison, under the same conditions, viral RNA was still detectable after 4 weeks (Fig. S2). When the mosquitoes were preserved in either ethanol or RNAlater, neither viral nor mosquito RNA showed major change in concentration over 4 weeks at room temperature.

### Follow-up phylogenetic studies

The taxon-specific primers used in the high-throughput sequencing assay were designed to amplify all members of the chosen group while avoiding off-target amplification and providing as much taxonomic information as possible. However, these criteria, combined with the requirement for short sequences (to be sequenceable on a massively parallel sequencer) sometimes limits their resolution.

**Fig. 3.**
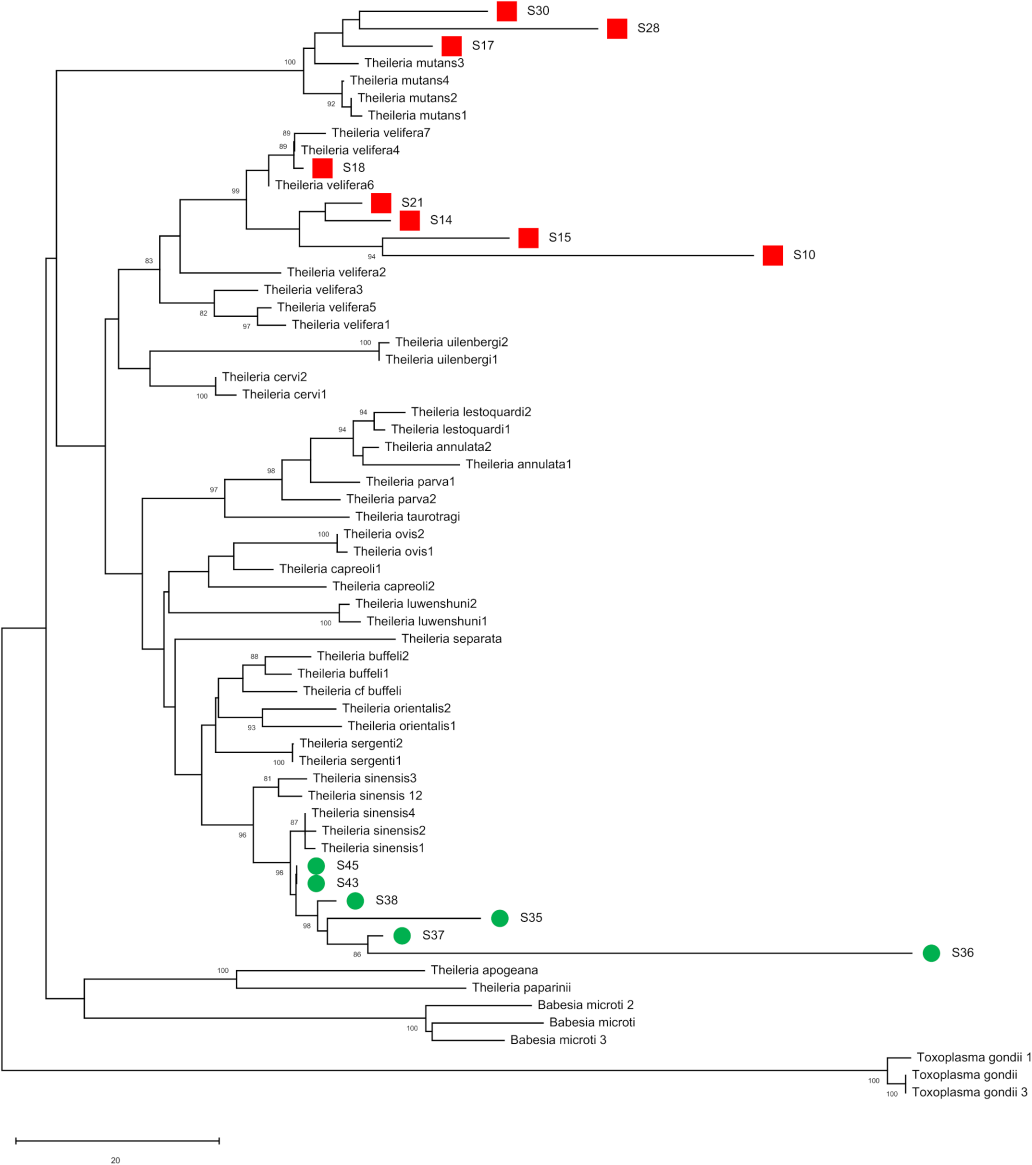
**Phylogenetic analysis of *Theileria* sequences amplified from Cambodian and African *Anopheles* mosquitoes.** The neighbor-joining tree shows the relationships between the 18S rRNA *Theileria* sequences amplified from samples positive by high-throughput sequences and those from known *Theileria* species deposited in NCBI. Sequences amplified from Cambodian mosquitoes are indicated in green circles, those amplified from African mosquitoes in red squares. Alignment is available as Dataset 2.

Thus, the Apicomplexa primers amplified multiple *Theileria* sequences but did not distinguish among species. We therefore amplified a longer DNA sequence (900 bp) of the 18S rRNA locus from the *Theileria*-positive African and Cambodian mosquitoes and sequenced them using Sanger sequencing technology. Phylogenetic analysis of these longer sequences, together with known *Theileria* species sequences deposited in NCBI, showed that the parasites amplified from the Cambodian mosquitoes were closely related to *T. sinensis*, while those from African mosquitoes were most closely related to *T. velifera* and *T. mutans* (Fig. 3).

**Fig. 4.**
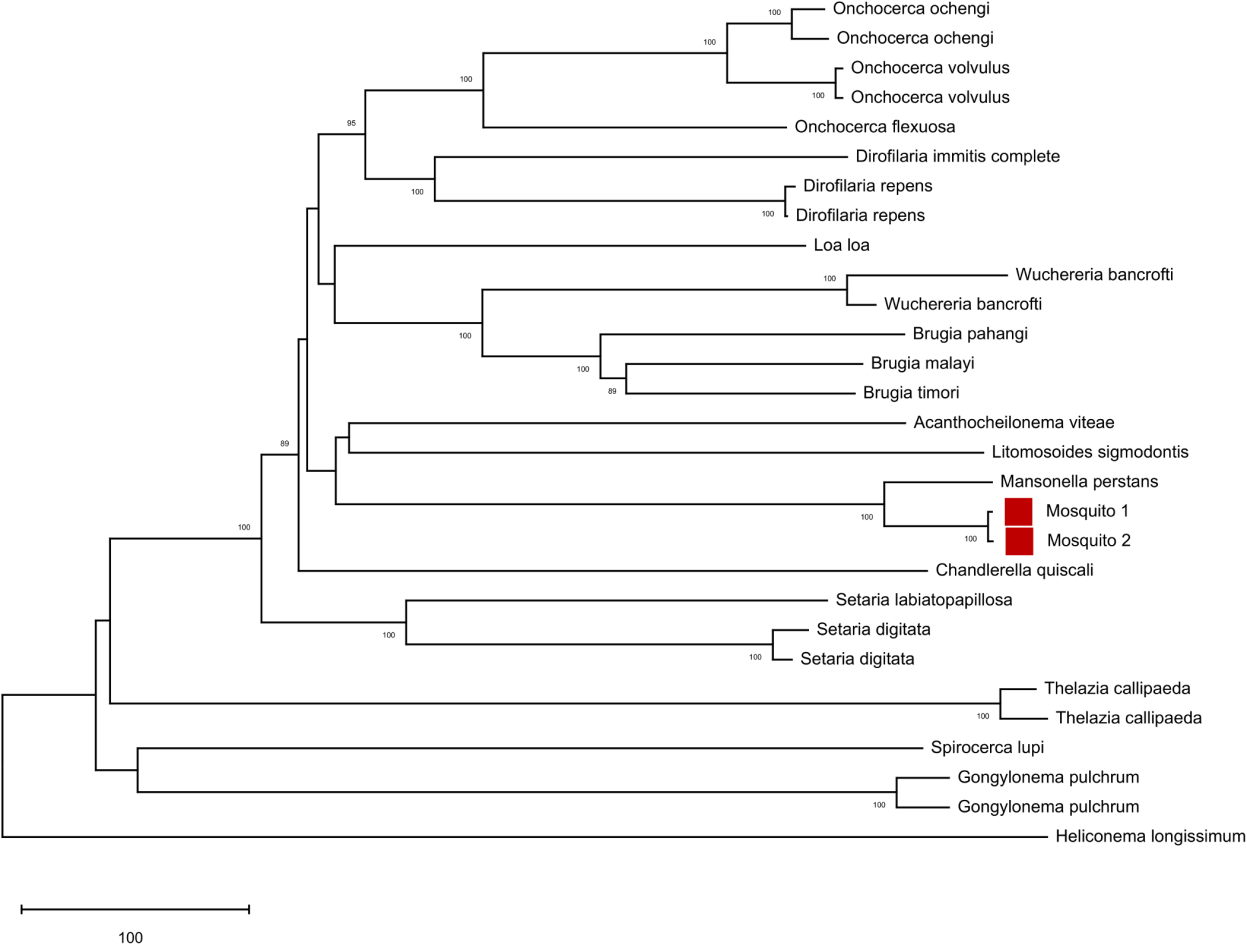
**Phylogenetic analysis of unknown filarial worm sequences amplified from Guinean mosquitoes.** The neighbor-joining tree shows the relationships between annotated filarial worm sequences and a 3.5 kb sequence amplified from two African mosquitoes (red squares) positive for filarial worms and sequenced using PacBio chemistry. Alignment is available as Dataset 3.

We also detected, in several African mosquitoes, filarial worm sequences whose taxonomic assignment was uncertain. One sequence was 100% identical to both *Loa loa* and *Dipetalonema sp. YQ-2006* (also known as *Mansonella*), while the sequence obtained from the same mosquitoes using a different primer pair was also most similar to *Dipetalonema* (*Mansonella*) but with 99.2% identity. To clarify the taxonomy of these sequences, we used PacBio long read technology to sequence 3.5 kb of filarial worm mitochondrial DNA (amplified by long range PCR from these two mosquitoes). We compared these sequences to known filarial worm mitochondrial DNA sequences and found that these were most similar to, but distinct from, *Mansonella perstans* (94 and 96 nucleotide differences or ∼97.0% identity), while *L. loa* was much more distantly related (∼83.3% identity) (Fig. 4). The genetic distance between *M. perstans* and *L. loa* in this tree was much higher (519 nucleotide differences, 83.5% identity) than using short amplicon data where these two sequences were identical, providing greater confidence in the phylogenetic analysis. We concluded that the filarial worms were most likely either *M. perstans* or a very closely related species.

### Analysis of individual versus pooled mosquitoes

We analyzed both individual mosquitoes and pools of 50–291 mosquitoes. For the pools, 23 out of the 25 produced sequences demonstrating that the amplification of pools of up to 291 mosquitoes is feasible without significant PCR inhibition. Out of the 930 individual mosquitoes, 162 (17.4%) had at least ten reads from one or more parasites or arboviruses. By comparison, 23 out of the 25 traps (92%) yielded such sequences, suggesting that analysis of pools might be an efficient way to screen for rare parasites (although this possibility would need to be rigorously evaluated in future studies as the individual mosquitoes and the CDC traps were not collected from the same geographic locations in our study and the species represented in the pools differed from those of the individual mosquitoes).

### Analysis of DNA versus RNA extracted from the same mosquito traps

All primers used for detecting parasites and viruses are located within single-exon genes and can amplify either DNA or cDNA with the same efficiency. However, the PCRs target genes that are typically highly expressed (e.g. ribosomal RNA genes) and we would therefore expect many more copies of RNA than DNA per cell (although this could be balanced by the faster degradation of RNA molecules compared to DNA). To evaluate the relative sensitivity of our assay for screening DNA and RNA, we compared the results obtained by analyzing matched DNA and RNA isolated from the same mosquito pools. We found that for Spirurida, Kinetoplast, Microsporidia and *Plasmodium* PCR assays, 62.2% of the sequences identified were detected only in the cDNA sample and not in the corresponding DNA sample from the same trap. For cases where a sequence was detected in both cDNA and DNA from a given trap, the cDNA yielded more reads in 89 of 119 instances (with, on average, 24.5-fold more reads). Read counts were higher in the DNA for only 29 of 119 cases with an average fold difference of 2.8 (one sample yielded equal read counts in cDNA and DNA). Out of these 29 cases, 22 (75.9%) of the sequences were most similar to *Trypanosoma* species despite these sequences representing only 17.4 of all sequences. On average, the cDNA samples produced 258–1169 more reads per hit than the matching DNA samples (Fig. 4), despite the storage of the samples at room temperature without preservative for more than 24 h.

## DISCUSSION

Vector-borne disease surveillance is an essential component of infectious disease control as it can enable rapid detection of outbreaks and guide targeted elimination efforts (e.g. through insecticide spraying). However, current approaches are extremely demanding with regards to human and financial resources, both for the sample collection and the identification of potential pathogens. Consequently, public health officials and vector biologists often have to focus on a handful of parasites associated with the most current threats. Current detection approaches also often lead to duplicated efforts, as different agencies interested in specific pathogens perform sample collection independently and have a high risk of failing to detect emerging pathogens until they cause outbreaks. Here, we describe application of a genomic assay that allows identification of a wide range of pathogens that can cause human and animal diseases, as well as of parasites of the vector (which could potentially be useful as biological controls).

**Fig. 5.**
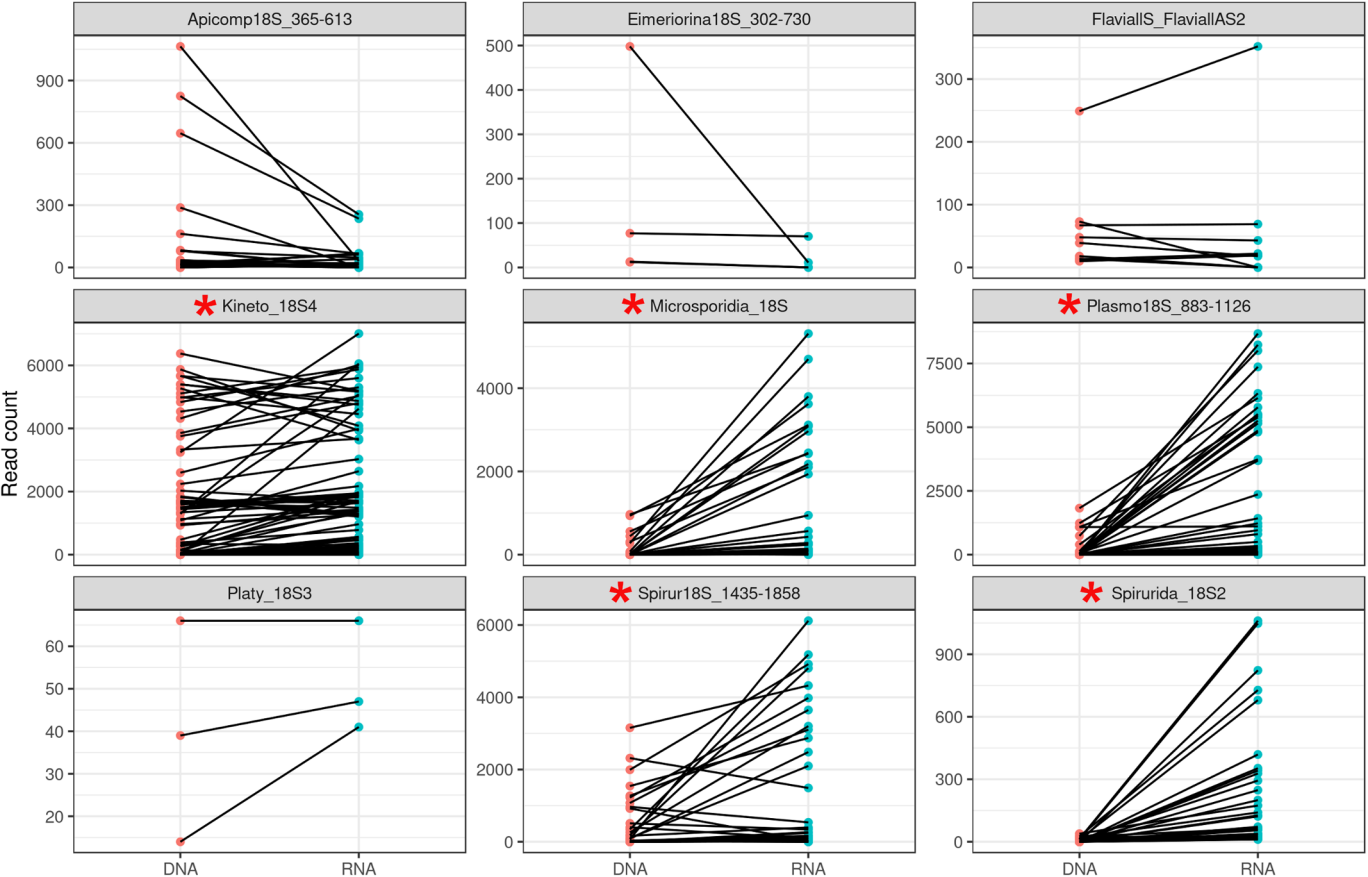
**Comparison of the number of reads obtained for different taxa from matched DNA and cDNA samples derived from Maryland mosquito pools.** Each panel represents results from one primer set and each pair of points connected by a line shows the number of reads matching a single species detected in both the DNA (left) and RNA (right) from the same sample. For five primers (red asterisks), the RNA samples yield significantly more reads than the matching DNA samples (*P*<0.05, Bonferroni-corrected pairwise *t*-tests).

The analyses of several hundred mosquitoes collected in Cambodia, Mali, Guinea and the USA revealed well-known human pathogens including *P. falciparum*, which was the target of the initial study of the Cambodian samples (Laurent et al., 2016)*.* In addition, we detected *Theileria* species and *Setaria digitata*, which cause livestock diseases in Southeast Asia (Nakano et al., 2007; Weerasooriya et al., 2016; Bawm et al., 2014; Liu et al., 2017; Shin et al., 2002). While we were initially unable to conclusively determine the exact *Theileria* species with our initial assay, targeted follow-up studies using longer amplicons and Sanger sequencing (Fig. 2) revealed that the sequences amplified from the African mosquitoes were most closely related to *T. velifera* and *T. mutans*, which are both known to infect African cattle (Mans et al., 2015), whereas the Cambodian mosquitoes carried sequences most closely related to *T. sinensis*, a species that infects cattle in China (Liu et al., 2010). *Theileria* parasites are transmitted by ticks, not mosquitoes, and the DNA sequences recovered likely derive from parasites taken up by the mosquitoes during a blood meal but likely not transmissible to another hosts. The *Schistosoma* species detected in mosquitoes from Africa also likely result from parasites present in a bloodmeal. In this regard, it is interesting to note that when one considers the samples collected in Maryland and analyzed with both DNA and RNA, the read counts (a proxy for the abundance of extracted molecules) for transmissible parasites (e.g. *Plasmodium*) or parasites of the mosquitoes (e.g. *Crithidia*, *Strigomonas* and *Takaokaspora*) were typically higher in the RNA samples than in the matched DNA samples while the opposite was true for parasites ‘sampled’ during the blood meal but unlikely to develop in *Anopheles* mosquitoes (e.g. *Theileria*, *Trypanosoma*) (Fig. 5; Fig. S3 and Table S3). We speculate that this difference is due to the difference between developing, live, parasites still synthesizing RNA molecules and dead (possibly digested) parasites for which the RNA is slowly being degraded. Comparison of DNA and RNA from the same mosquito could perhaps provide a tool to differentiate transmissible parasites from those sampled by the vector (although adequate sample preservation would be necessary as RNA is typically much more rapidly degraded than DNA).

We also identified, in two African mosquitoes, sequences similar to known filarial worms but identical to multiple sequences present in the database. Using this information, we characterized longer DNA sequences and showed that these two mosquitoes likely carried *M. perstans* parasites. Since the PCR primers were designed to amplify any member of the selected taxa, they can reveal the presence of novel pathogens as long as they are phylogenetically related to known parasites. This feature is a key advantage of our assay for vector-borne disease surveillance as it may enable early detection of emerging pathogens and zoonoses and provide a basis for rapid response.

In addition to known human parasites and potential emerging pathogens, this single-stop assay also provides another source of information valuable for vector-borne disease control: 9% of the individual mosquitoes and 62% of pooled mosquito samples screened yielded sequences of microsporidians related to well-characterized arthropod parasites, which could potentially be used to guide the development of targeted biological vector control. This ability to detect multiple parasites at once in a high-throughput manner and across a wide range of taxonomical groups could reduce duplication of collection efforts and costs, as mosquitoes collected for one purpose could be screened for many parasites affecting both humans and animals. In addition, characterization of a wide range of parasites present in a given mosquito may also improve our understanding of the general factors regulating infection and transmission: several studies have shown that immunity and previous infections can influence the response of mosquitoes to human parasites and their transmission (Cirimotich et al., 2010; Bian et al., 2013; Meister et al., 2005) and information of current infections of wild-caught mosquitoes could, for example, significantly improve our assessment of their vector capacity.

Several of the infectious diseases that have recently caused major public health challenges by spreading outside of their typical range (Higuera and Ramirez, 2019; Sejvar, 2016) or emerging as novel human infectious diseases (Gorshkov et al., 2018; Liu et al., 2019), are caused by viruses transmitted by mosquitoes. We therefore extended our assay to capture, using the same approach as for eukaryotic parasites, both known and novel flaviviruses. Since flaviviruses are RNA viruses and RNA degrades much faster than DNA, we first examined how nucleic acid degradation influenced our ability to detect virus over time. To test RNA preservation, we collected mosquitoes known to carry *Culex flavivirus* and isolated RNA from pools of five mosquitoes, either immediately frozen or kept at room temperature for 2 or 4 weeks, with either no preservative, ethanol or RNAlater. The mosquitoes stored in preservatives had minimal loss of viral (and mosquito) RNAs as determined by qRT-PCR (Fig. S2). Even when stored without preservatives, viral RNA were detectable after 4 weeks at room temperature (although with a reduction of, on average, 10.7 PCR cycles), demonstrating a remarkable stability of the RNA, possibly due to protection provided by the viral capsid (by contrast very little mosquito RNA remained amplifiable after 2 weeks at room temperature, Fig. S2). As a proof-of-principle and to demonstrate the potential of this approach for viral disease surveillance, we screened the Maryland mosquito pools and the individual African mosquitoes for flaviviruses. We identified several viruses, distinct from known viruses (Fig. 2) and, based on their phylogenic position, likely to infect mosquitoes rather than humans. Alternatively, these sequences could represent polymorphic viral integrations into the mosquito genome (Palatini et al., 2017; Whitfield et al., 2017; Russo et al., 2019; Pischedda et al., 2021).

Based on the results described above, we believe that this single high-throughput assay can provide a wide range of information critical for vector-borne disease researchers and public health officials. However, several limitations need to be noted. One caveat is that, whereas false positive detection of a species is highly unlikely (aside from laboratory cross-contamination), several factors could lead to false negatives. Thus, while the primers were designed to amplify all known sequences of a given taxon as effectively as possible, nucleotide differences at the primer binding sites could prevent efficient amplification of a specific species. This potential problem could be particularly problematic if several related parasites are present in the same sample but are differentially amplified: for example, it could be possible that a *Plasmodium* parasite might be mis-detected if the sequences generated by an Apicomplexan primer pair are out competed by *Theileria* sequences. Similarly, poor preservation of the nucleic acids in one sample could also lead to false negatives. False negatives could also occur for stochastic reasons: if only a few parasite cells are present in one sample (e.g. an *Anopheles* mosquito infected by a *Plasmodium* ookinete) it is possible that no DNA will be present in the PCR reaction (especially if the extract gets divided across many reactions). One approach to circumvent this limitation could be to test cDNA instead of DNA (Kamau et al., 2011; Adams et al., 2015): our analyses of the Maryland mosquitoes showed that, for many primer sets, amplification of cDNA resulted in higher read counts than amplification of DNA extracted from the same samples, despite the sub-optimal preservation of these samples. Another limitation is the specificity of taxonomic assignment. As discussed above, if the sequenced amplicon does not contain enough information to distinguish similar species, subsequent experiments may be required to confirm pathogen identity. A particularly problematic case would be if an emerging parasite with no reference sequence is identical at the sequenced locus to a known human pathogen. In this case, the analysis could lead to an unambiguous, and incorrect, assignment of the sequence to the wrong pathogen. While rare, the potential for taxonomical mis-assignment highlights the importance of following up on unexpected or important findings with additional verification experiments. Alternatively, the use of several loci for identification could prevent such cases since the different informativity of each locus could reveal discrepancies (as we observed for the filarial worm detected in the African mosquitoes).

Finally, we showed that analyses of fairly large pools of mosquitoes (up to ∼300 mosquitoes) were possible with our assay. This feature could be extremely useful in specific situations where only a small fraction of all mosquitoes are expected to carry the pathogen of interest, although the exact gain in sensitivity of analyzing pools would need to be rigorously evaluate in future studies (since the dilution of the pathogen DNA by pooling and the increase competition with related pathogens during the amplification could negatively impact the sensitivity). Overall, this assay provides a customizable method to screen, in a high-throughput and cost-efficient manner, hundreds of samples for a wide range of potential parasites and, as such, can complement existing methods for vector-disease surveillance: while many approaches have been successfully developed to test many samples for the presence of a particular organism in a very sensitive and specific manner, there are currently very few assays enabling to simultaneously screen for many organisms, including uncharacterized species. An alternative approach to detect and characterize all parasites present in one sample uses shotgun sequencing (or metagenomics): all DNA or RNA molecules are extracted from the chosen sample and directly sequenced before bioinformatic analysis. Since the nucleic acids are (typically) not selected, there are little biases introduced and molecules from all organisms should be sequenced (circumventing possible mis-amplification during the PCR). Additionally, since the sequences derive from the entire genome (as opposed to a single locus), the taxonomic assignment is greatly facilitated and mis-classification unlikely. However, since pathogen nucleic acids only represent a fraction of the DNA/RNA present in the sample, extensive sequencing is required to detect them (Greninger, 2018; Chiu and Miller, 2019; Carpi et al., 2015) leading to a high cost and a low throughput. Selectively enriching for pathogen nucleic acids prior to sequencing may circumvent partially this limitation (but see also Carpi et al., 2015) but re-introduces biases in the detection. A better study design could perhaps combine the best of both approaches: screening a very large number of samples using the assay described here and selecting the few potentially interesting samples for shotgun sequencing.

### Conclusion

This study demonstrates how a high-throughput, one-stop assay could efficiently complement current toolkits to prevent vector-borne diseases by providing a broad description of known and emerging human viruses and parasites, informing on animal pathogens that could affect a region's economy, and indicating possible biological control candidates that could be used against these disease vectors. One additional feature of this sequencing-based assay is the ease of customizing it to different settings and research questions. Since the assay relies on PCR primers, it is straightforward to add and remove primers for specific taxa of interest, or to combine them with additional PCRs to characterize, for example, the source of the blood meal (Logue et al., 2016).

## MATERIALS AND METHODS

### Samples

We analyzed a total of 930 individual mosquitoes, as well as 25 pools each containing 50–291 mosquitoes (2589 total) (Table 2; Tables S4–S6).

**Table 2.**
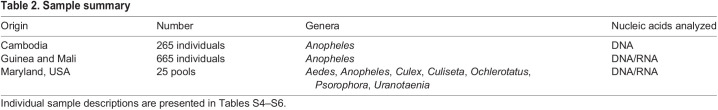
Sample summary

First, we analyzed DNA previously extracted from 265 individual *Anopheles* mosquitoes collected in the Cambodian provinces of Pursat, Preah Vihear, and Ratanakiri (Laurent et al., 2016). These mosquitoes were collected using cow- or human-baited tents, human landing collections, CDC light traps and barrier-screen fences and immediately preserved by desiccation upon collection. These 265 *Anopheles* mosquitoes represent 22 different species collected between July and August of 2013 (see Table S4 for details).

Second, we included DNA samples from 81 individual mosquitoes collected in Bandiagara, Mali. DNA from these samples was extracted using Chelex^®^ 100 (Bio-Rad) after incubation of bisected and homogenized mosquitoes in 1% saponin in PBS.

Third, we extracted DNA from 584 individual *Anopheles* mosquitoes collected in six sites in Guinea and preserved in ethanol immediately upon collection. These mosquitoes were collected by human landing catch and pyrethrum spray (Table S5). Each mosquito was homogenized in 200 µl ATL/proteinase K solution using five RNase-free 1 mm zirconium oxide beads in a TissueLyser II for 12 min at 20 m/s. We centrifuged the solution at 2500 rpm for 3 min and incubated them at 55°C for 1 h. We performed a second homogenization step for four minutes at 20 m/s followed by a final incubation at 55°C overnight. We then isolated DNA using the Qiagen DNeasy 96 Blood & Tissue Kit according to the manufacturer's instruction and eluted DNA from each sample in 200 µl.

Finally, we analyzed 25 pools of mosquitoes collected throughout Prince George's county (Maryland, USA) by the Maryland Department of Agriculture using CO_2_-baited light traps (Table S6). Each pool contains all mosquitoes from one light-trap (∼50–291 mosquitoes) and was stored at room temperature for up to 24 h before long-term storage at −20°C. We homogenized each pool of mosquitoes using a Qiagen TissueLyser II with Teenprep Matrix D 15 ml homogenization tubes (MP Biomedicals) and isolated successively RNA and DNA from each sample using the RNeasy PowerSoil Total RNA kit (Qiagen) with the RNeasy PowerSoil DNA Elution Kit and a final elution volume of 100 µl.

### Evaluation of Arbovirus primers

We tested universal flavivirus primers retrieved from the literature (Patel et al., 2013) on West Nile (*n*=3), Zika (*n*=2) and Dengue (*n*=2) viral RNAs obtained from the American Type Culture Collection (ATCC). We synthesized cDNA from 2 µL of RNA using M-MLV reverse transcriptase (Promega) and random hexamers, and amplified the resulting cDNA with GoTaq^®^ DNA polymerase (Promega) under the following conditions: initial two-minute denaturing step at 95°C followed by 40 cycles of 95°C for 30 s, 50°C for 30 s and 72°C for 40 s. A final extension at 72°C for 10 min was followed by incubation at 4°C. We ran the products on an agarose gel to determine whether each virus RNA was amplifiable.

### PCR amplification of pathogen nucleic acids before high-throughput sequencing

First, we synthesized cDNA using M-MLV reverse transcriptase (Promega) and random hexamers from either (1) 2 µl of the nucleic acids isolated from the Guinean mosquitoes (i.e. using RNA carried over during the DNA extraction), or (2) 3 µl of RNA extracted from the pools of Maryland mosquitoes.

Then, we amplified DNA and cDNA (when available) separately from each sample, as well as from 176 no-DNA controls, with a total of 11 primer pairs, each targeting a specific taxon known to contain human pathogens (Table S1). For each primer pair, we amplified DNA and cDNA using GoTaq^®^ DNA polymerase (Promega) under the following conditions: initial denaturing step at 95°C followed by 40 cycles of 95°C for 30 s, 50°C for 30 s and 72°C for 30 s. A final extension at 72°C for 10 min was followed by incubation at 4°C. All primers used in these taxon-specific PCRs included 5′-end tails to serve as priming sites for a second PCR. We then pooled all PCR products generated from one sample and performed a second PCR using primers targeting these tails to incorporate, at the end of each amplified molecule, (i) a unique oligonucleotide ‘barcode’ specific to each sample and (ii) DNA sequences complementary to the Illumina sequencing primers (Cannon et al., 2016; Logue et al., 2016) (Fig. 1). For the Maryland pools, DNA and cDNA derived from a single biological sample were barcoded separately to allow comparisons. cDNA from African mosquitoes was used only to amplify flaviviruses and was pooled with the DNA amplified reactions prior to barcoding. Finally, we pooled together the resulting barcoded libraries and sequenced them on an Illumina sequencer to generate an average of 12,703 paired-end reads of 251 or 301 bp per sample.

### Bioinformatic analyses

We first separated the reads generated from each sample according to their unique barcodes and merged the overlapping ends of each read pair using PANDAseq (Masella et al., 2012) to generate consensus DNA sequences and correct sequencing errors (that disproportionally occur at the end of the reads). Note that all primers were designed to amplify DNA sequences shorter than ∼450 bp, allowing overlap of at least 50 bp between paired-end reads. All read pairs that did not merge correctly were discarded from further analyses. We identified and trimmed the primer sequences from each read and eliminated all consensus sequences shorter than 100 bp as they likely represent experimental artefacts (e.g. PCR chimeras and primer dimers). Using reads from all samples together, we recorded how many unique DNA sequences were obtained and how many reads carried each of these unique DNA sequences. Sequences observed less than ten times in the entire dataset were omitted as they likely resulted from PCR or sequencing errors (Cannon et al., 2016). We then compared each unique DNA sequence to all sequences deposited in the NCBI nt database using BLAST (Altschul et al., 1990) and used custom pipelines (https://github.com/MVesuviusC/2020MosquitoSurveillancePaper) to retrieve the taxonomic information associated with the most similar sequence(s). For each sample, only sequences with at least 10 reads and more than 70% identity with an annotated NCBI sequence over the entire sequence length were further considered. This low identity cutoff allows inclusion of results from highly genetically divergent organisms which can then be examined further (see below). This is critical when DNA is amplified from species without closely related sequences available. If DNA sequences from multiple species were equally similar to one of our sequences, we recorded all corresponding species names. Finally, we summarized, for each mosquito or pool, the parasite species or virus identified, the percentage identity between the reads and the most similar NCBI sequence(s), and the number of reads supporting the identification in this sample.

### Phylogenetic analyses

To better characterize specific DNA sequences with ambiguous species identification, we analyzed these sequences together with orthologous sequences from closely related species. Briefly, we used PrimerTree (Cannon et al., 2016) to retrieve NCBI orthologous DNA sequences from all species of the targeted taxon. We aligned these sequences with the DNA sequence(s) amplified from the mosquito(es) using MAFFT (Katoh and Standley, 2013) and reconstructed neighbor-joining trees using MEGA (Tamura et al., 2013) to estimate the phylogenetic position of the amplified DNA sequences.

### Further determination of taxonomical assignments

To improve species identification when multiple species had identical DNA sequences, or improve phylogenetic analyses of unknown sequences, we amplified and sequenced specifically chosen DNA loci from pathogens using DNA from the mosquitoes carrying these sequences.

For differentiating *Theileria* species, we used previously published primers (GGCGGCGTTTATTAGACC, TCAATTCCTTTAAGTTTCAGCC [31]) to amplify an informative portion of the 18S rRNA gene using DNA from 19 samples identified as *Theileria* positive by high-throughput sequencing. Amplification was conducted under the following conditions: initial denaturing step at 95°C for 2 min followed by 40 cycles of 95°C for 30 s, 50°C for 30 s and 72°C for 40 s. A final extension at 72°C for 5 min was followed by incubation at 4°C. Since gel electrophoresis revealed multiple bands, we used a Pasteur pipette to collect a core from the agarose gel, corresponding to the expected 900 bp PCR product, and dissolved it in 100 µl of water at 60°C for 20 min. We then re-amplified 10 µl of this DNA using 35 PCR cycles with the same conditions. After gel electrophoresis, we treated the PCR reaction with 0.046 µl of Exonuclease I (NEB) and 0.4625 µl of Shrimp alkaline phosphatase (Affymetrix) at 37°C for 30 min, with a final five-minute inactivation step at 95°C. We then Sanger sequenced each PCR product in both directions using the forward and reverse primers. We manually trimmed the reads and merged them using Flash (Magoc and Salzberg, 2011). We aligned the reads, along with known *Theileria* sequences from the NCBI nucleotide database, using MAFFT (Katoh and Standley, 2013; Katoh et al., 2017) and generated a neighbor joining tree with 500 bootstraps and plotted it in MEGA7 (Kumar et al., 2016).

To identify the species of the filarial worms detected in two individual mosquitoes, we designed primers to amplify a 3.5 kb portion of the mitochondrial DNA. Briefly, we downloaded all available filarial worm (Filarioidea) mitochondrial sequences from the NCBI nucleotide database, aligned them, generated a consensus sequence and designed primers using primer3 (Untergasser et al., 2012). We then used these primers (TTCGTCGTGAGACAGAGCGG, AGGCCATTGACGGATGGTTTGTAC) to amplify DNA from the two positive mosquitoes using the Expand™ Long Range dNTPack kit (Sigma-Aldrich) using the following conditions: initial denaturing step at 95°C for 2 min followed by 45 cycles of 92°C for 30 s, 55°C for 30 s and 68°C for 5 min. A final extension at 68°C for 10 min was followed by incubation at 4°C. We then performed a second PCR to add 10 bp barcodes to the 5′ end of both forward and reverse primers to allow differentiating both samples after sequencing. The two barcodes differed by 8 and 7 bases for the forward and reverse primers, respectively, with no more than two identical bases in a row (Table S1). For this second PCR, we used the following conditions: initial denaturing step at 95°C for 2 min followed by ten cycles of 92°C for 30 s, 55°C for 30 s and 68°C for 5 min. A final extension at 68°C for ten minutes was followed by incubation at 4°C. We purified the amplicons using AMPure XP beads (Beckman Coulter) (2:1 DNA:beads ratio) and then combined equimolar amounts of each barcoded PCR product before circular consensus sequencing on a PacBio Sequel. We then generated a consensus sequence for each sample and aligned these sequences to known nematode mitochondrial sequences using Mafft (Katoh and Standley, 2013) and generated a neighbor joining tree in MEGA (Kumar et al., 2018).

### Assessment of the dynamics of viral and mosquito RNA degradation

To assess the dynamics of viral RNA degradation over time, we analyzed *Culex pipiens* mosquitoes from a laboratory colony known to be infected with *Culex flavivirus*. The colony was initiated from diapausing adult *C. pipiens* that were collected from Oak Lawn and Des Plaines, IL, USA, on 2/8/10. These two collections were combined to make one colony, which was determined to be *C. flavivirus* positive by reverse transcriptase PCR (Patel et al., 2013). We examined RNA preservation in mosquitoes stored with no preservative, in ethanol or in RNAlater (Invitrogen). Three pools of five mosquitoes were analyzed for each condition and at each time point (i.e. fresh, after 2-week or after 4-week storage at room temperature). After 0, 2 or 4 weeks at room temperature, the mosquitoes were stored at −80°C until RNA isolation. We isolated RNA from each pool of mosquitoes using Qiazol (Qiagen) and eluted into 50 µl. We synthesized cDNA from 7 µl of RNA using m-MLV (Promega) with random hexamers for PCRs using *Culex* primers and, separately, on 2 µl of RNA for PCRs using flavivirus primers.

For each pool of five *Culex* mosquitoes from the *C. flavivirus*-infected colony, we performed quantitative reverse transcriptase PCR (qRT-PCR) to quantify the amount of mosquito and viral RNAs using the primers Culex_flavivirus_3F (TGCGAARGATCTDGAAGGAG) - Culex_flavivirus_3R (CACGCACAACAAGACGATRA) targeting the virus sequence, and Culicinae_Cox1_379_F (AYCCHCCTCTTTCATCTGGA) - Culicidae_Cox1_670_R (CCTCCTCCAATTGGRTCAAAG) targeting transcripts from the mosquito Cox1 gene. We used Perfecta SYBR green PCR mastermix (Quantabio) with the following conditions: initial 15-min denaturing step at 95°C followed by 40 cycles of 95°C for 30 s, 55°C (Culex primers) or 50°C (flavivirus primers) for 30 s and 72°C for 1 min (Culex primers) or 40 s (flavivirus primers). We performed standard cycle threshold and melt curve analysis afterwards using default settings.
